# Role of Metabolic Syndrome Traits on Infectious Diseases: A Mendelian Randomization Study

**DOI:** 10.3389/ijph.2025.1607945

**Published:** 2025-12-03

**Authors:** Si Cao, Youjie Zeng, Xiaoyi Zhang, Juan Tang, Jie Huang, Guoxin Lin

**Affiliations:** 1 Department of Anesthesiology, Third Xiangya Hospital, Central South University, Changsha, China; 2 Department of Medicine, Jacobi Medical Center, Albert Einstein College of Medicine, Bronx, NY, United States; 3 Department of Nephrology, Third Xiangya Hospital, Central South University, Critical Kidney Disease Research Center of Central South University, Changsha, China; 4 Clinical Research Center for Reproduction and Genetics in Hunan Province, Reproductive and Genetic Hospital of CITIC-XIANGYA, Changsha, China

**Keywords:** causal inference, metabolic syndrome, COVID, bacterial pneumonia, intestinal infection

## Abstract

**Objectives:**

To explore the causal association of metabolic syndrome (MetS) and its components [systolic blood pressure (SBP), fasting blood glucose (FG), waist circumference (WC), high-density lipoprotein cholesterol (HDL-C), and triglycerides (TG)] with seven infectious diseases (COVID-19 infection, hospitalized COVID-19, very severe COVID-19, bacterial pneumonia, influenza, intestinal infection, and sepsis) using Mendelian randomization (MR) analysis.

**Methods:**

Causal estimates were primarily obtained using the inverse-variance weighted method, with multiple sensitivity analyses conducted to assess heterogeneity and horizontal pleiotropy.

**Results:**

MetS was causally associated with higher risks of COVID-19 infection (OR = 1.09), hospitalized COVID-19 (OR = 1.27), very severe COVID-19 (OR = 1.40), and sepsis (OR = 1.50). Among MetS components, WC increased risks of COVID-19 infection (OR = 1.10), hospitalized COVID-19 (OR = 1.39), very severe COVID-19 (OR = 1.56), bacterial pneumonia (OR = 1.11), and sepsis (OR = 1.42), while HDL-C reduced risks of intestinal infection (OR = 0.96) and sepsis (OR = 0.92).

**Conclusion:**

This MR study supports a causal link between MetS traits and several infectious diseases, emphasizing the importance of metabolic management in reducing infection susceptibility.

## Introduction

Infectious diseases are major causes of global morbidity and mortality, affecting hundreds of millions of people worldwide annually [1, 2]. Severe infections, which can rapidly evolve into sepsis, multi-organ failure, and even death, account for more than 20% of global deaths [3]. Infectious diseases that commonly require hospitalization include viral infections (Coronavirus disease 2019, Influenza), bacterial pneumonia, intestinal infections, and sepsis. Identifying the underlying risk factors for these infectious diseases is crucial for improving global public health.

Metabolic syndrome (MetS) encompasses a range of metabolic disorders, including central obesity, high blood pressure, elevated blood glucose levels, increased triglyceride (TG) levels, and reduced high-density lipoprotein cholesterol (HDL-C) levels. MetS prevalence exhibits a steady global increase, ranging from 20% to 50%, with severely obese adolescents reaching 50% [4, 5]. MetS significantly increases the likelihood of developing diabetes, stroke, and cardiovascular diseases [6]. Numerous studies have suggested that metabolic syndrome and its associated conditions, including obesity, diabetes, and fatty liver diseases, are linked to a heightened risk of several infectious diseases [7]. For instance, pre-existing metabolic dysfunction, such as obesity, hypertension, and diabetes, has been identified as exacerbating the course of COVID-19, and high blood glucose levels and fluctuating blood glucose levels may unfavorably impact COVID-19 outcomes [8]. It is important to acknowledge that the relationship between MetS and infectious diseases might be affected by confounders, limited sample sizes, and constrained follow-up duration, thus yielding inconclusive findings.

Mendelian randomization (MR) is an approach that uses genetic variations as instrumental variables (IVs) to estimate the causal association between exposure and outcome. Compared to traditional observational studies, MR studies minimize confounding factors and reverse causation as genetic variation arises during meiosis [9]. Numerous genome-wide association studies (GWAS) and the corresponding summary-level datasets provide the feasibility of conducting MR studies. Here, we conducted a bidirectional two-sample MR strategy to ascertain the causal associations between MetS (including its components) and various infectious diseases.

## Methods

### Research Design

**FIGURE 1:**
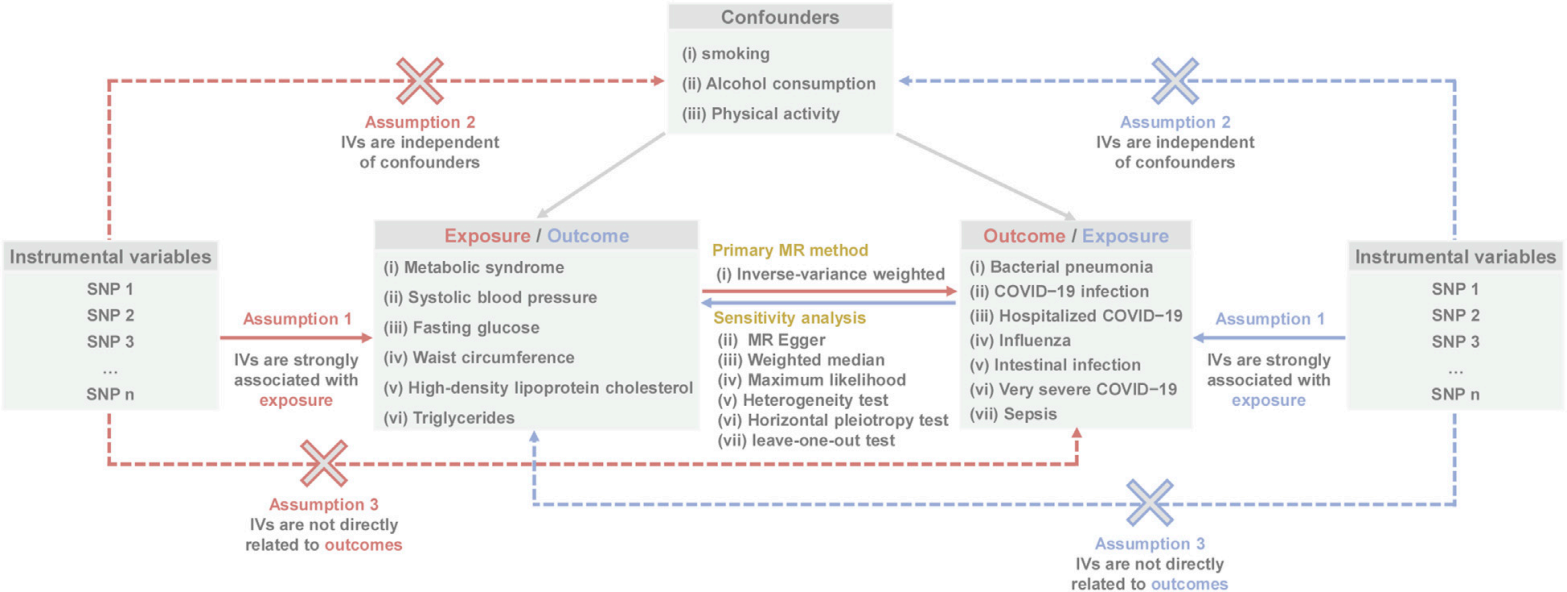
Overall analysis flow of this study (Europe, 2018–2024).

Figure 1 illustrates the overall flow chart of the present MR study. Specifically, the GWAS summary-level statistics for MetS, five MetS components, and seven infectious disease traits [(i) bacterial pneumonia; (ii) COVID-19 infection; (iii) hospitalized COVID-19; (iv) influenza; (v) intestinal infection; (vi) very severe COVID-19; and (vii) sepsis] were initially downloaded. Subsequently, causal effects of MetS and five MetS components on seven infectious diseases were estimated by conducting two-sample MR analyses. Then, the causal effects of seven infectious disease traits on MetS and five MetS components were assessed by reverse MR analysis. Finally, diverse sensitivity tests were conducted on the significant estimation results to assess the reliability. This study was conducted using the “TwoSampleMR” package in R software.

### Data Sources for MetS and MetS Components

GWAS summary datasets for MetS were derived from a recent study by van Walree et al. [10], which included 461,920 individuals of European ancestry and identified the most comprehensive set of MetS-associated variants to date. Additionally, we accessed GWAS summary-level statistics for five key MetS components: systolic blood pressure (SBP), fasting glucose (FG), waist circumference (WC), HDL-C, and TG. All the GWAS datasets were based exclusively on European individuals. Comprehensive details of these datasets are provided in Supplementary Table S1.

### Data Sources for Seven Infectious Disease Traits

GWAS summary data for COVID-19 were obtained from the COVID-19 Host Genetics Initiative (Release 7) [11], including: (i) COVID-19 infection (122,616 cases vs. 2,475,240 controls), (ii) COVID-19 necessitating hospitalization (32,519 cases vs.2,062,805 controls), and (iii) critical COVID-19 cases, characterized by severe outcomes (13,769 cases vs.1,072,442 controls). Summary datasets of bacterial pneumonia (17,511 cases vs. 344,010 controls), influenza (9204 cases vs. 344,010 controls), and intestinal infection (44,967 cases vs. 367,214 controls) were obtained from the FinnGen database (Version R10; https://www.finngen.fi/en/access_results). Summary dataset of sepsis was obtained from the IEU OpenGWAS database (id: ieu-b-4980; https://gwas.mrcieu.ac.uk/). Detailed dataset information is listed in Supplementary Table S1.

### Selection of IVs

IVs for conducting MR analysis were selected according to three core MR assumptions: (i) SNPs were strongly correlated with exposure; (ii) SNPs were not related to confounding factors; (iii) SNPs showed no direct correlation with the outcome [9]. To satisfy the first assumption, using a genome-wide significance threshold (*P* < 5e-8), SNPs significantly associated with the exposures were first selected. In addition, SNPs in linkage disequilibrium (r^2^ < 0.001 within a 10,000 kb window) were excluded to ensure independence. To fulfill the second core assumption, SNPs linked to potential confounding factors (smoking, alcohol consumption, and physical activity) were excluded. Supplementary Table S2 shows the sources of GWAS summary statistics for confounders. Supplementary Table S3 presents details of SNPs associated with confounding factors. For the third core assumption, SNPs potentially associated with COVID-19 traits (*P* < 0.05) were excluded from the IVs. Then, palindromic SNPs were excluded. Moreover, the *F*-statistics of each IV were assessed. To minimize bias from weak IVs, only those with *F*-statistics exceeding 10 were selected. In addition, if the amount of IVs obtained for reverse MR analysis based on the *P* < 5e-8 threshold was too limited, the threshold was relaxed (*P* < 1e-5 or *P* < 5e-5) depending on the number of available IVs. Furthermore, the number of SNPs varied among different GWAS summary-level statistics. Therefore, for reverse MR analysis, prior to screening IVs, only the common SNPs between the GWAS summary statistics for seven infectious disease traits and the GWAS statistics for MetS/MetS components were retained.

### Statistical Analysis

The main MR approach for causal inference was inverse variance weighted (IVW). IVW first assessed the causal effect of exposure on outcome using the Wald ratio for each individual IV, followed by a meta-analysis by fixed or random effects models [12]. In this study, a random-effects IVW model was applied to account for potential heterogeneity across genetic instruments and to provide more conservative estimates. A *P*-value of less than 0.05 was regarded as statistically significant. As the outcomes were binary traits, MR results were presented using odds ratios (OR) with 95% confidence intervals (CI). Various sensitivity tests were conducted to validate the significant findings derived from the main analysis. First, three supplementary MR methods (MR-Egger, weighted median, and maximum likelihood method) were performed. Subsequently, Cochran’s Q test was conducted to determine heterogeneity. Then, the horizontal pleiotropy was assessed using the MR-Egger intercept MR-PRESSO global test. Finally, the leave-one-out test was performed to determine whether there were abnormal leading SNPs that significantly influenced the overall results.

## Results

### Results of Forward MR Analysis

This section presents the estimated causal effects of MetS and the five MetS components (SBP, FG, WC, HDL-C, and TG) on seven infectious disease traits. Details of IVs used for forward MR analysis are shown in Supplementary Table S4, with *F*-statistics of ranging from 25 to 5621, indicating adequate instrument strength.

**FIGURE 2:**
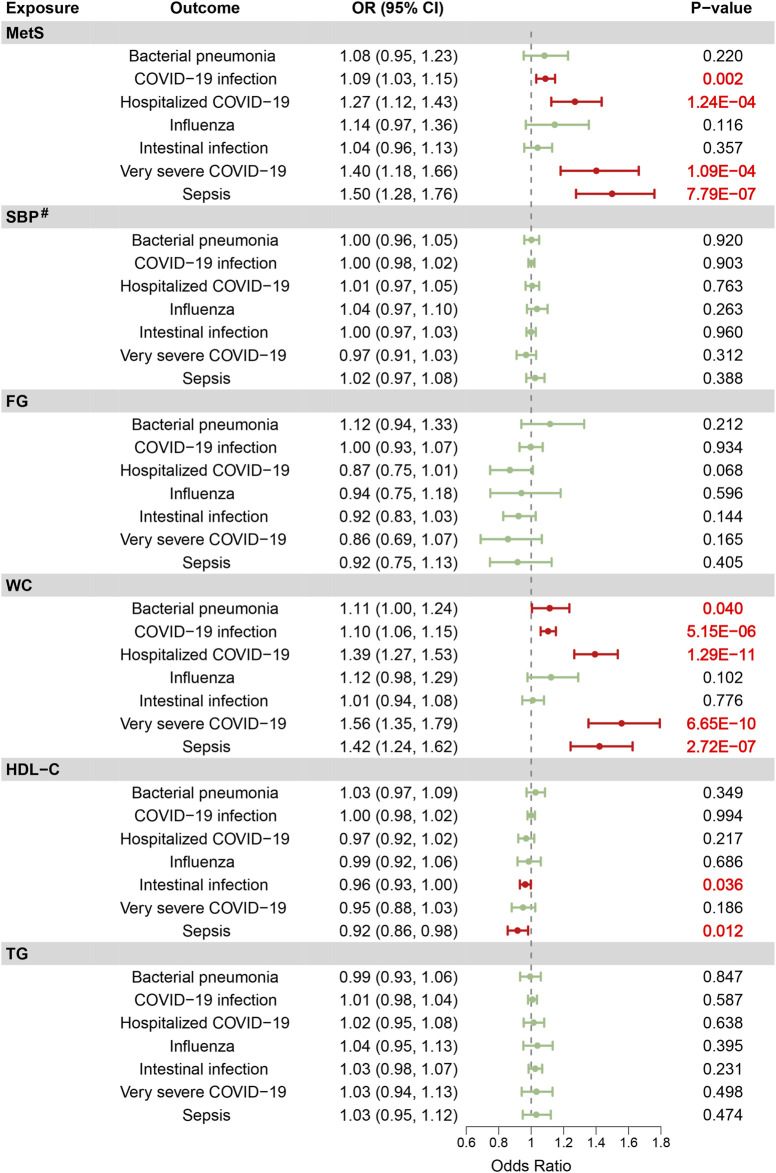
Identification of the causal effects of metabolic syndrome and its five components on seven infectious disease traits by inverse-variance weighted Mendelian randomization method (Europe, 2018–2024). ^#^ Odds ratios for SBP represent the effect per 10 mmHg increase.

The IVW analysis indicated that MetS was significantly associated with an increased risk of COVID-19 infection (OR = 1.09, 95% CI: 1.03–1.15, *P* = 0.002), hospitalized COVID-19 (OR = 1.27, 95% CI: 1.12–1.43, *P* = 1.24E-04), very severe COVID-19 (OR = 1.40, 95% CI: 1.18–1.66, *P* = 1.09E-04), and sepsis (OR = 1.50, 95% CI: 1.28–1.76, *P* = 7.79E-07) (Figure 2). Among the MetS components, WC showed a significant positive causal relationship with bacterial pneumonia (OR = 1.11, 95% CI: 1.00–1.24, *P* = 0.040), COVID-19 infection (OR = 1.10, 95% CI: 1.06–1.15, *P* = 5.15E-06), hospitalized COVID-19 (OR = 1.39, 95% CI: 1.27–1.53, *P* = 1.29E-11), very severe COVID-19 (OR = 1.56, 95% CI: 1.35–1.79, *P* = 6.65E-10), and sepsis (OR = 1.42, 95% CI: 1.24–1.62, *P* = 2.72E-07). In addition, HDL-C was negatively associated with intestinal infection (OR = 0.96, 95% CI: 0.93–1.00, *P* = 0.036) and sepsis (OR = 0.92, 95% CI: 0.86–0.98, *P* = 0.012).

Sensitivity analyses supported the robustness of these associations. Firstly, four additional MR approaches (MR-Egger, weighted median, and maximum likelihood) exhibit parallel findings to IVW (OR > 1 for MetS and WC & OR < 1 for HDL-C) (Supplementary Table S5). Secondly, no heterogeneity was identified by Cochran’s Q test (*P* > 0.05) (Supplementary Table S6). Thirdly, no remarkable horizontal pleiotropy was detected by the MR-Egger intercept test and MR-PRESSO global test (*P* > 0.05) (Supplementary Table S6). Finally, the leave-one-out analysis confirmed the robustness of the results, as excluding any single IV did not lead to significant changes in the findings (Supplementary Figures).

### Results of Reverse MR Analysis

This section presents the estimated causal effects of genetic liability to seven infectious disease traits on MetS and MetS components. Detailed information on IVs used for reverse MR analysis is shown in Supplementary Table S7, with *F*-statistics ranging from 16 to 845. However, MR analysis by the IVW method indicated that genetic predisposition to these infectious disease traits did not causally contribute to MetS and MetS components (*P* > 0.05) (Supplementary Table S8).

## Discussion

The present study employed a bidirectional MR approach to scrutinize the causal association between MetS and its components and various infectious diseases. Our findings unveiled that MetS and WC increased the likelihood of three phenotypes of COVID-19 and sepsis; WC increased the risk of bacterial pneumonia; HDL cholesterol decreased the incidence of intestinal infection and sepsis. However, no causal relationships were observed between FG, TG, systolic blood pressure, and these infectious diseases. Reverse MR analysis revealed that genetic liability to infectious diseases infectious diseases did not appear to causally affect MetS and its components.

A retrospective cross-sectional study involving American adults has revealed that individuals with MetS experience significantly higher hospitalization and mortality rates due to COVID-19, even after controlling for covariates [13]. A meta-analysis has further suggested that MetS is linked to a 2.3-fold higher risk of short-term mortality due to COVID-19 [14]. Findings from an observational study demonstrated that obesity, diabetes, and a history of stroke are associated with a greater likelihood of death from COVID-19 in comparison to individuals who succumb to non-COVID-19 causes [15]. Notably, up to 50% of COVID-19 fatalities have been found to possess underlying metabolic and vascular disorders [16]. The presence of age-related comorbidities, like hypertension, diabetes, obesity, and cardiovascular diseases, which are prevalent among older individuals (≥65 years), significantly impacts the progression and prognosis of COVID-19 [17]. Using a Mendelian randomization approach, our study demonstrated that MetS could potentially elevate COVID-19 infection risk, hospitalization rate, and disease severity, thereby reinforcing previous observational findings.

WC, as a constituent of MetS, has demonstrated superior efficacy as an anthropometric measure for assessing visceral adipose tissue mass and serves as a reliable indicator of abdominal obesity [18]. Given the pivotal role of obesity in MetS and its significant association with COVID-19, it is imperative to explore this relationship further. Raeisi et al. recently reported that obesity escalates the likelihood of contracting COVID-19, progressing to severe illness, necessitating hospitalization, admission to intensive care units, and even death [19]. Similarly, Brenda et al. evaluated obesity-related traits in COVID-19 patients and found that BMI and WC were associated with severe respiratory COVID-19 and hospitalized COVID-19 [20], which is consistent with our results. Moreover, an additional study revealed a J-shaped relationship between BMI and the risk of COVID-19 severity and mortality [21]. However, a prospective observational study indicated that, compared to individuals with modest COVID-19, individuals with severe COVID-19 exhibited elevated FG and TG levels, while no statistically significant variations were identified in WC, BMI, systolic blood pressure, and HDL-C levels [22]. This discrepancy may be attributed to a smaller sample size and other confounding factors.

In general, this study first indicated that MetS increased the incidence and severity of COVID-19. Subsequently, causality between individual components of MetS and COVID-19 was estimated, highlighting the critical role of WC. Although previous observational studies have reported associations between SBP, FG, HDL-C, TG, and COVID-19, this MR study suggests no causal association between these four MetS components and COVID-19. There are several potential mechanisms that could explain the role of WC being the most essential component of MetS that contributes to COVID-19 progression. Studies have posited that the presence of abdominal obesity correlates with augmented deposition of visceral adipose tissue, potentially facilitating the progression toward severe manifestations of COVID-19 [23]. Adipose tissue, an active metabolic organ, exhibits hormonal and cytokine secretion [24]. In the setting of obesity, dysfunctional adipose tissue triggers the release of pro-inflammatory cytokines, including interleukin-6, tumor necrosis factor-α, and C-reactive protein [25]. Such cytokines have the potential to contribute to cytokine storms in COVID-19, thereby intensifying the severity of the disease and causing harm to various organs, including the lungs [26]. Furthermore, adipose tissue exhibits a pronounced upregulation of ACE-2 receptors, which serve as the entry point for the SARS-CoV-2 virus. The elevated presence of ACE-2 receptors may result in a heightened viral load and consequently exacerbate the severity of COVID-19 in Obese individuals [27]. Additionally, obesity has been recognized as a contributing factor for endothelial dysfunction, magnifying the preexisting impairment of endothelial function induced by COVID-19 [28]. Consequently, this pathological cascade may culminate in the formation of blood clots, precipitating severe complications including stroke and pulmonary embolism [29].

Additionally, multiple studies revealed that obesity, a key component of metabolic syndrome, doubles the likelihood of developing influenza and is associated with increased severity of influenza [30]. Nevertheless, our findings showed a trend toward a positive but non-significant association between MetS, WC, SBP, TG, and influenza. These relationships should be explored further using future GWAS datasets with larger sample sizes.

The present study showed that MetS and WC were linked to an increased sepsis risk, and elevated WC was related to a higher risk of bacterial pneumonia. Consistently, previous studies have reported metabolic syndrome and increased waist circumference as significant risk factors for sepsis [31]. A population-based cohort investigation in the USA showed WC as a better predictor of sepsis risk than BMI [31]. Patients with abdominal obesity were at a 1.74-fold greater risk for sepsis mortality [32]. Metabolic syndrome is linked to chronic low-grade inflammation, which may impair immune function and increase susceptibility to severe bacterial infections [33]. Moreover, studies have suggested that patients hospitalized with community-acquired bacterial pneumonia frequently have abdominal obesity [34]. The excess belly fat in abdominal obesity can pose pressure on the stomach, contributing to gastroesophageal reflux, which is a risk factor for aspiration pneumonia, a common cause of sepsis [35].

In this study, we also identified a remarkable relationship between elevated HDL-C and decreased risks of intestinal infection and sepsis. This finding is consistent with prior studies. HDL-C has been found to regulate innate and adaptive immunity, impacting the immune response to infections [36]. Studies indicate that HDL-C levels reduce rapidly at the onset of sepsis, and low serum HDL-C levels are linked to poor outcomes [37]. Low HDL-C levels are indicative of increased severity of septic diseases [38], and are linked to an amplified systemic inflammatory response [39]. HDL, alongside various plasma lipids, demonstrates a remarkable ability to interact with and counteract components such as Gram-negative bacterial lipopolysaccharide and Gram-positive bacterial lipoteichoic acid [40]. This interaction is beneficial in promoting the removal of these bacterial products from the system. However, exceedingly high HDL-C levels may also be linked to a greater risk of infectious disease, potentially due to genetic variants or functional impairment of HDL particles [41].

This study has several strengths. Firstly, multiple phenotypes of both exposure and outcome were included, increasing the comprehensiveness of the research. Secondly, the two-sample MR study utilized various large-scale GWAS summary datasets, thereby increasing the robustness of the findings. Thirdly, multiple sensitivity analyses were employed to enhance the credibility of the MR causal estimates.

There are certain limitations in this MR study that require clarification. First, since all the GWAS data included were from individuals of European descent, it remained uncertain whether the conclusions could be generalized to other populations. Second, because this MR study was based on summary-level GWAS statistics, it was not possible to conduct stratified analyses by age or gender. Lastly, although extensive sensitivity tests were performed, it was not possible to completely rule out the influence of potential horizontal pleiotropy.

### Conclusion

In conclusion, this study strengthens the observed correlation between MetS and infectious diseases. The present findings emphasize the need for improved management of MetS, especially among obese patients with larger waist circumference or low HDL-C levels, to reduce the future incidence of infectious diseases.

## Data Availability

Information of the summary datasets for MR analysis are detailed in Supplementary Table S1.

## Abbreviations

MetS, Metabolic syndrome; TG, triglyceride; HDL-C, high-density lipoprotein cholesterol; MR, Mendelian randomization; IV, instrumental variable; GWAS, genome-wide association studies; SBP, systolic blood pressure; FG, fasting glucose; WC, waist circumference; IVW, inverse variance weighted; OR, odds ratios; CI, confidence intervals.
