# Supplementary material for: Role of Metabolic Syndrome Traits on Infectious Diseases: A Mendelian Randomization Study
Source: Int J Public Health. 2025 Dec 3;70:1607945. doi: 10.3389/ijph.2025.1607945 (PMC12708360; doi:10.3389/ijph.2025.1607945)
Supplement: Supplementary file 2 [file DataSheet1.pdf]

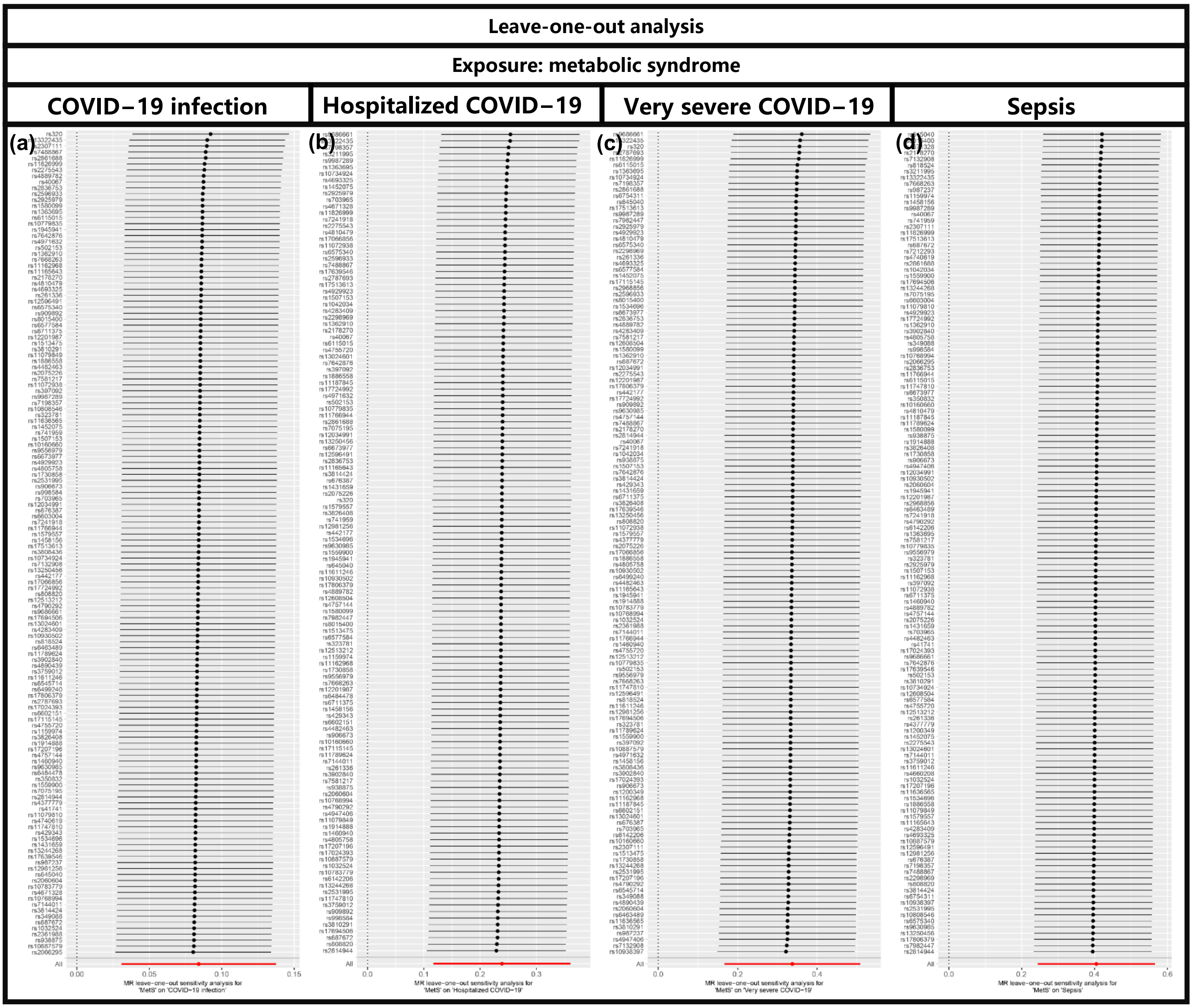

Figure S1. MR leave−one−out sensitivity analysis for "metabolic syndrome" on **(a)** "COVID−19 infection", **(b)** "hospitalized COVID−19", **(c)** "very severe COVID−19", and **(d)** "sepsis".

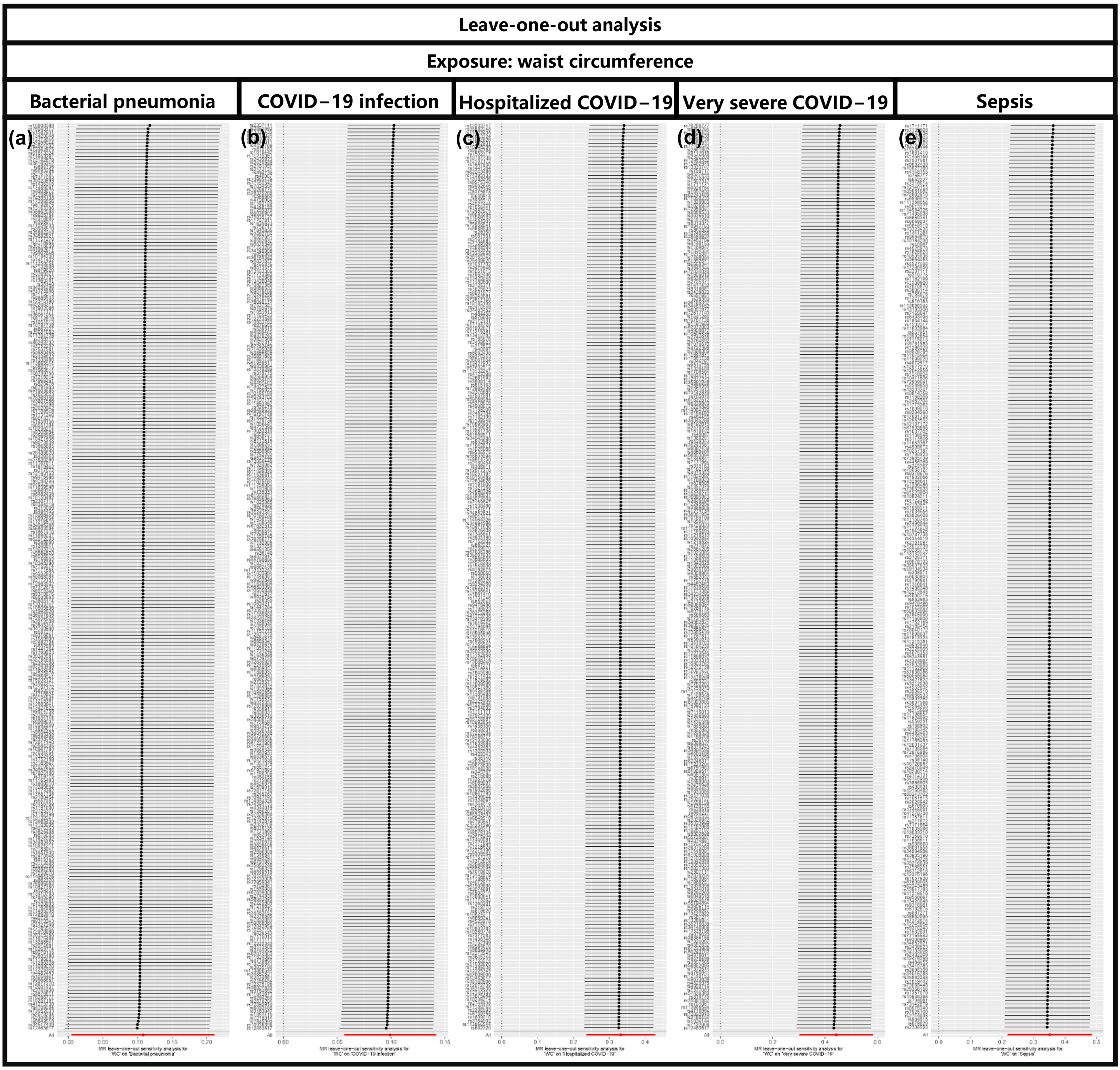

Figure S2. MR leave−one−out sensitivity analysis for "waist circumference" on **(a)** "bacterial pneumonia", **(b)** "COVID−19 infection", **(c)** "hospitalized COVID−19", **(d)** "very severe COVID−19", and **(e)** "sepsis".

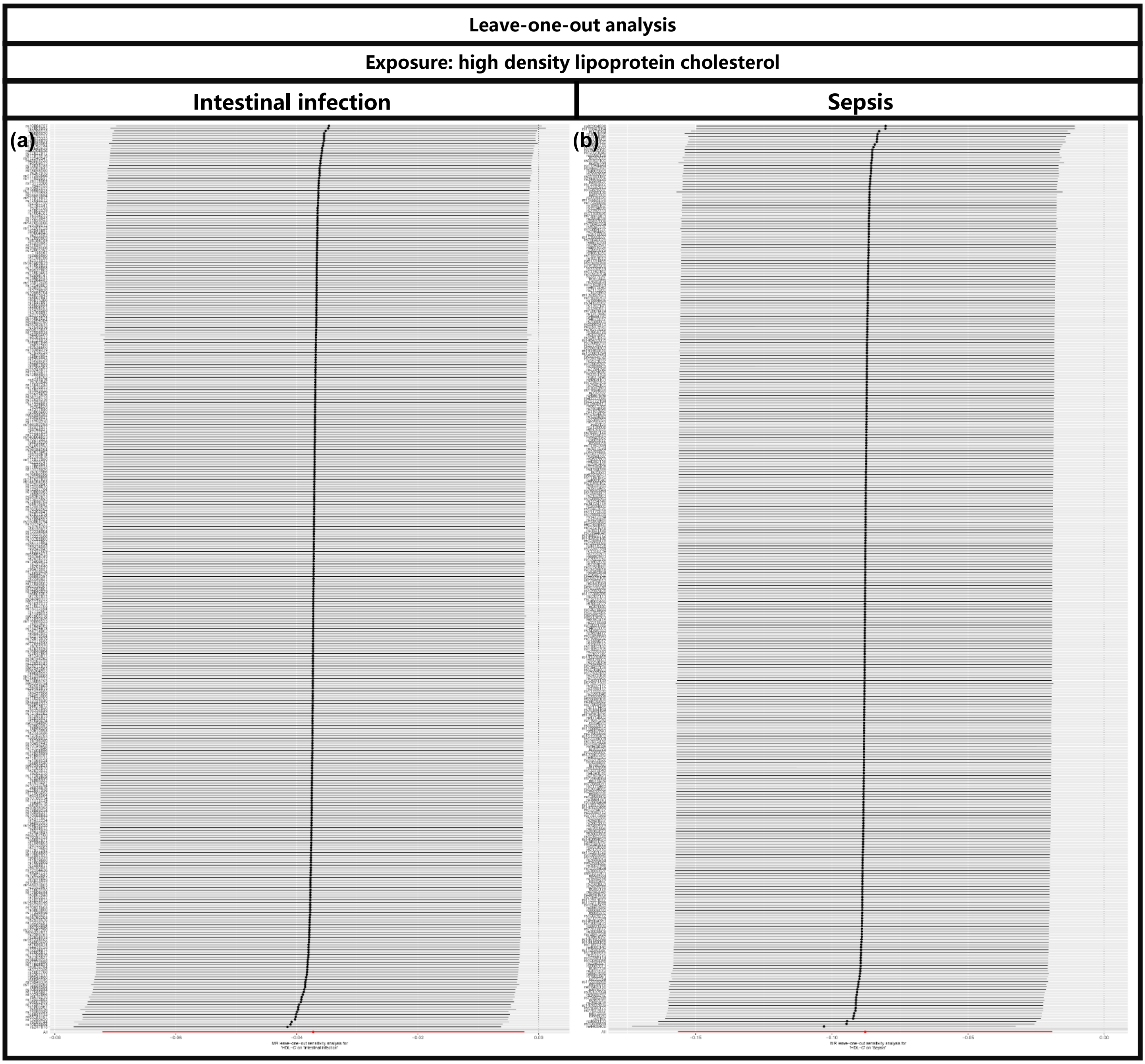

Figure S3. MR leave−one−out sensitivity analysis for "high density lipoprotein cholesterol" on **(a)** "intestinal infection" and **(b)** "sepsis".
